# Pathogenesis and neurotropism of a contemporary Powassan virus lineage II strain in mice

**DOI:** 10.1128/jvi.00585-26

**Published:** 2026-08-31

**Authors:** Samantha J. Courtney, Emily N. Gallichotte, Emma Nilsson, Chasity E. Trammell, Kate X. Kimball, Anna C. Fagre, Allison C. Vilander, Anna K. Överby, Gregory D. Ebel

**Affiliations:** 1 Department of Microbiology, Immunology and Pathology, Colorado State University 3447, Fort Collins, Colorado, USA; 2 Department of Clinical Microbiology, Umeå University 8075, Umeå, Sweden; 3 Laboratory for molecular infection medicine Sweden (MIMS), Umeå University 8075, Umeå, Sweden; The University of Texas Southwestern Medical Center, Dallas, Texas, USA

**Keywords:** viral determinants, tick-borne flavivirus, neuroinvasion, pathogenesis, Powassan virus

## Abstract

Powassan virus (POWV) is an emerging tick-borne flavivirus that can cause neurologic disease in humans. POWV exhibits substantial genetic and phenotypic diversity, including marked variability in replication *in vitro* and pathogenesis in mice. However, most experimental studies have relied on historical lineage I and II strains to assess POWV pathogenesis. Here, we characterized a contemporary lineage II strain, NY.19.12, in mice and investigated the potential viral determinants of disease. NY.19.12 caused earlier onset of clinical signs and earlier detection of viral RNA (vRNA) in the spleen and brain compared to mice infected with a widely used Midwest lineage II strain (WI.97.ic). Sequencing of NY.19.12 identified three amino acid differences in the envelope, non-structural 1, and non-structural 5 proteins. These substitutions are also present in other lineage II strains currently circulating in the Northeast United States, suggesting that they are maintained in nature. To assess their contribution to pathogenesis, the three amino acid substitutions were engineered into an infectious clone and evaluated in mice. At early time points post-infection, clinical signs and viral distribution in the brain were similar between NY.19.12 and the mutant clone, suggesting that these amino acid substitutions may contribute to disease progression and neurotropism. However, NY.19.12 vRNA was detected in the spleen at significantly higher rates than both WI.97.ic and the mutant clone, indicating that factors beyond these mutations contribute to persistence in the spleen. Together, these findings highlight the complexity of POWV pathogenesis and suggest that lineage II strains exhibit variable disease phenotypes likely driven by multiple genetic determinants.

**IMPORTANCE:**

Tick-borne flaviviruses exhibit considerable genetic and phenotypic diversity in nature, but it is unclear if this influences their transmission and pathogenesis. Defining the mechanisms of pathogenesis requires understanding how inter-lineage variation translates to phenotypic differences in viral dissemination and neuroinvasion. This study demonstrates that even closely related Powassan virus (POWV) lineage II strains display distinct disease phenotypes that are only partially attributable to nonsynonymous consensus mutations within the viral coding sequence. By highlighting the complexity of strain-dependent POWV pathogenesis and neurotropism in mice, these findings provide valuable insights into how POWV lineage II diversity may shape disease progression and severity in humans.

## INTRODUCTION

Powassan virus (POWV, Flaviviridae, *Orthoflavivirus*) is a tick-borne *Orthoflavivirus* that causes severe neuroinvasive disease in humans (1). POWV is most frequently detected in the upper Midwest and Northeast United States, Canada, and the Russian Far East (2). POWV is classified into two distinct lineages. Lineage I appears to mainly be associated with *Ixodes cookei* and *Ixodes marxi*. Lineage II is mostly associated with *Ixodes scapularis*, which frequently feeds on humans (3, 4). POWV transmission in nature is highly focal, with genetically similar strains deriving from the same geographic region (5). There is limited understanding of virus strain-specific impacts on transmission by ticks and disease outcomes in vertebrates.

Following the bite of an infected tick, symptom onset in humans often begins with flu-like illness including fever and vomiting. In severe cases, symptoms progress to encephalitis characterized by headaches, confusion, loss of coordination, and speech difficulties (2, 6). In total, 10% of the clinically apparent cases are fatal, and neurologic sequelae persist in up to 50% of the symptomatic cases (7). There is an urgent need to better understand the pathogenesis of this emerging tick-borne virus because the number of reported human cases has markedly increased over the last 20 years (3, 8, 9).

C57BL/6 mice are widely used as an experimental model for POWV infection and pathogenesis, where peripheral inoculation routes such as subcutaneous (S.C.), intradermal, or footpad injections are designed to mimic natural tick-bite transmission. The virus replicates to high levels in the central nervous system (CNS) of 6- to 12-week-old C57BL/6 mice, leading to significant neuropathology and a high mortality rate (10–14). In addition, there is evidence that the virus accumulates in peripheral tissues such as the lymph nodes and spleen (15). Variation in pathogenesis within lineage I strains, and differences between lineage I and II have been documented (10, 13, 14, 16). However, the extent of variation in pathogenesis within the currently dominant lineage II, and the impact of specific viral determinants on pathogenesis remain unclear.

We thus sought to characterize the pathogenesis of a recently isolated, low-passage POWV lineage II strain, NY.19.12, isolated in 2019 from an *I. scapularis* tick collected in Saratoga County, New York. Although this strain has not been directly correlated to human POWV disease, Saratoga County has reported six confirmed human POWV cases between 2013 and 2023 (incidence rate: 0.24 per 100,000 population) and is classified as having moderate *I. scapularis* entomologic risk (3, 9). NY.19.12 is therefore an ecologically relevant strain that can inform our understanding of variation in regional POWV transmission and disease risk. Using C57BL/6 mice, morbidity, mortality, and tissue tropism were assessed between NY.19.12 and a historical lineage II infectious clone-derived virus. Significant variation in pathogenesis was observed, with NY.19.12 demonstrating higher rates of viral RNA (vRNA) detection in the spleen and brain. This strain and other POWV lineage II strains from the Northeast share three amino acid substitutions in the envelope protein (E), non-structural protein 1 (NS1), and non-structural protein 5 (NS5). When the amino acids were engineered into an infectious clone, they recapitulated the neurotropism of NY.19.12 but not spleen tropism. Overall, these results demonstrate that POWV pathogenesis is strain-dependent and highlight the complexity of genetic determinants of virus pathogenesis.

## RESULTS

### Selected POWV lineage II strains

**Fig 1:**
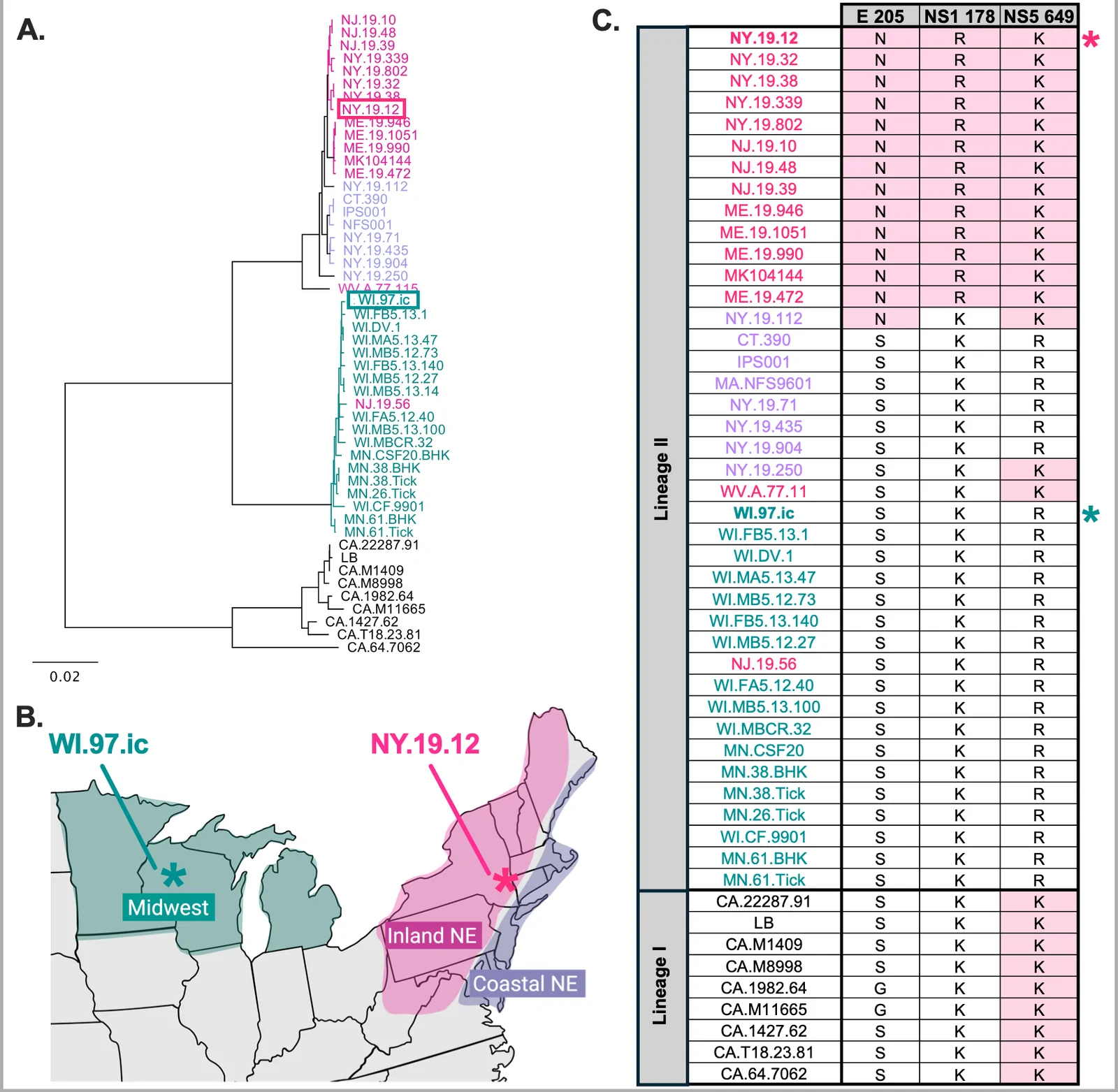
POWV lineage II strains and associated amino acid differences. (**A**) A rooted neighbor-joining phylogenetic tree of POWV lineage I and II complete polyprotein amino acid sequences was generated in Geneious Prime 2023.0.1 (black = lineage I; teal = lineage II, Midwest; purple = lineage II, coastal Northeast; pink = lineage II, inland Northeast). Strains used in subsequent studies are boxed. (**B**) U.S. map showing the location where each lineage II POWV strain was originally collected, color-coded as described for panel A (asterisks correspond to selected strains in the phylogenetic tree). (**C**) Amino acid residues at E-205, NS1-178, and NS5-649 among POWV lineage I and lineage II strains. Strains are ordered and colored based on the phylogenetic tree. Pink highlighted residues indicate the presence of the substitution (E-S205N, NS1-K178R, and NS5-R649K). Strains labeled with an asterisk (*) are viruses used in the study.

POWV lineage II strains group phylogenetically based on geographic origin (Fig. 1A) (5). NY.19.12 and WI.97.ic were selected to represent Northeast and Midwest lineage II strains, respectively, due to their ecological relevance to regional POWV transmission and disease risk (Fig. 1B, Table 1). WI.97.ic was additionally chosen to maintain consistency in subsequent genetic analyses due to the existence of a reverse genetics system based on this strain (17). Amino acid sequences of POWV lineage I and II strains were aligned, and it was determined that NY.19.12 and other Northeast lineage II strains share three amino acid substitutions in E, NS1, and NS5 proteins compared with other strains. The E and NS1 substitutions were only detected in lineage II, but the NS5 substitution was also detected in every lineage I strain examined (*n* = 50) (Fig. 1C).

**TABLE 1:** Characteristics of POWV lineage II strains used

Strain designation	Source location	Year of isolation	Species origin	Passage history^*a*^	Accession number
NY.19.12	Saratoga Springs, NY	2019	*I. scapularis*	B2	OP823461
WI.97.ic	Spooner, WI	1997	*I. scapularis*	B3	OP823482 ^*b*^

^*a*^ B, BHK cells.

^*b*^ WI.97.ic is a clone-derived virus from this GenBank accession number sequence.

### Viral tissue tropism in mice

**Fig 2:**
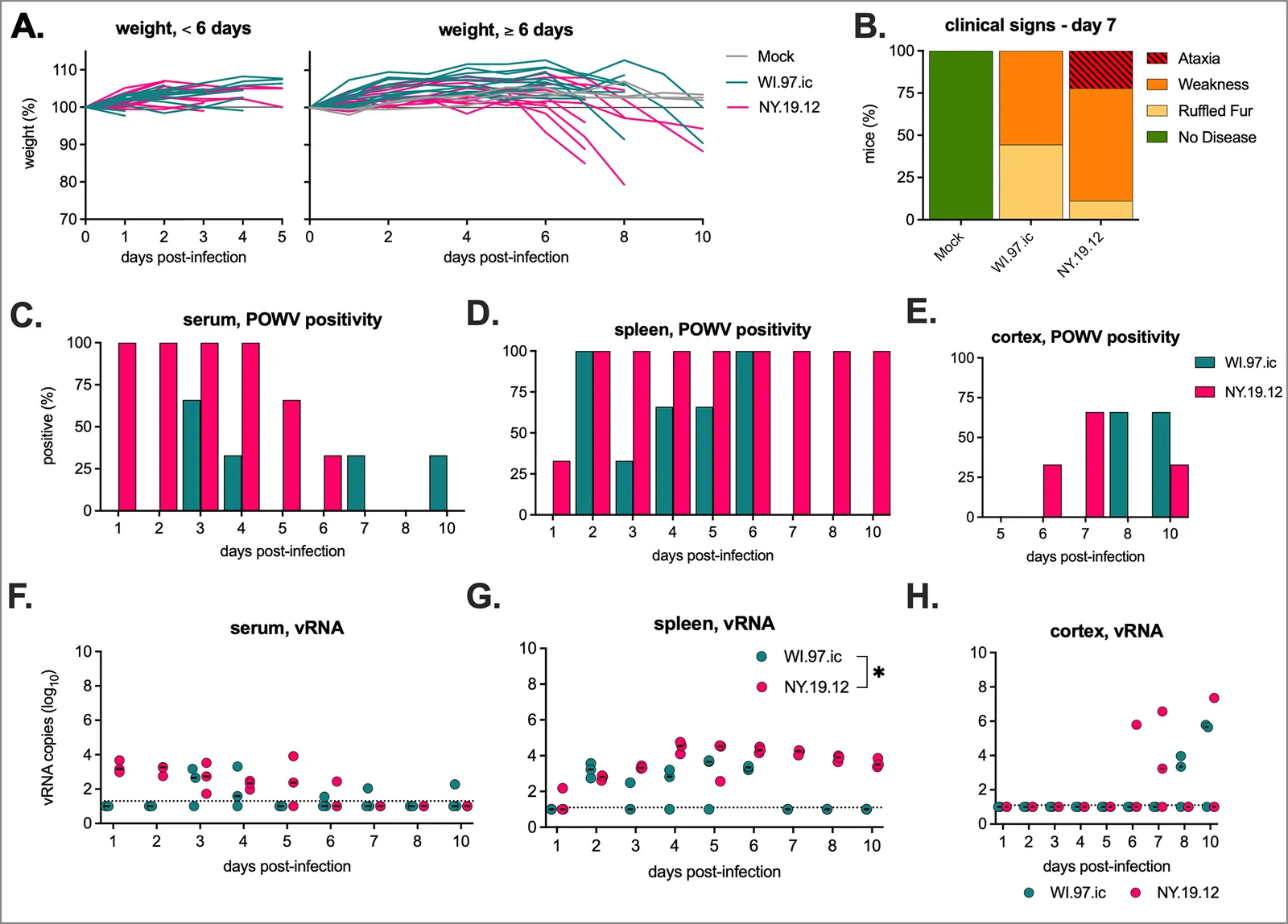
NY.19.12 displayed increased replication kinetics in mice. Mice were S.C. inoculated with 10^3^ PFU POWV (NY.19.12, WI.97.ic) or mock, sacrificed, and sampled over a 10-day course of infection (*n* = 27 per infection cohort, 3 mice sacrificed per time point). (**A**) Percent weight change from starting weight for mice sacrificed days 1 through 5 post-infection (*n* = 15) and those sacrificed after day 5 post-infection (*n* = 12), where each line represents individual mice until their designated harvest time point. (**B**) Clinical signs at 7 days post-infection (*n* = 9). Positive detection of POWV vRNA (%) in the (**C**) serum, (**D**) spleen, and (**E**) cortex per day. vRNA copies per (**F**) milliliter of serum or gram of (**G**) spleen or (**H**) cortex per day post-infection (median). The dotted line shows the limit of detection. For each sample type, comparisons of the total vRNA values for each strain across all timepoints were performed (Wilcoxon test, *P < 0.05). (mock = gray, pink = NY.19.12, teal = WI.97.ic).

To determine POWV replication kinetics and tissue tropism for NY.19.12 and WI.97.ic, C57BL/6 mice were S.C. inoculated and sampled over 10 days. Viral loads were assessed in the serum, spleen, and cortical tissue of the brain. For mice sacrificed between 1 and 6 days post-infection, there was no weight change (Fig. 2A). At 7 days post-infection, only mice infected with NY.19.12 had lost weight (Fig. 2A), and 15% met the endpoint criteria for this study (ataxia or 20% weight loss), compared to WI.97.ic-infected mice, which only displayed mild-to-moderate clinical signs (Fig. 2B). Viremia was detected more frequently in NY.19.12-infected mice compared to WI.97.ic at early time points (Fig. 2C). In addition, NY.19.12 vRNA was detected more frequently than WI.97.ic in spleens throughout the course of infection (Fig. 2D) and in the cortex at earlier time points (days 6 and 7) compared to WI.97.ic (day 8) (Fig. 2E). Despite lower rates of viremia and the presence of vRNA in the cortex at later time points in WI.97.ic-infected mice, the levels of vRNA in the serum and cortex were similar to NY.19.12 (Fig. 2F and H). In the spleen, however, NY.19.12-infected mice had significantly higher levels of vRNA compared to WI.97.ic (*P* < 0.05) (Fig. 2G).

**Fig 3:**
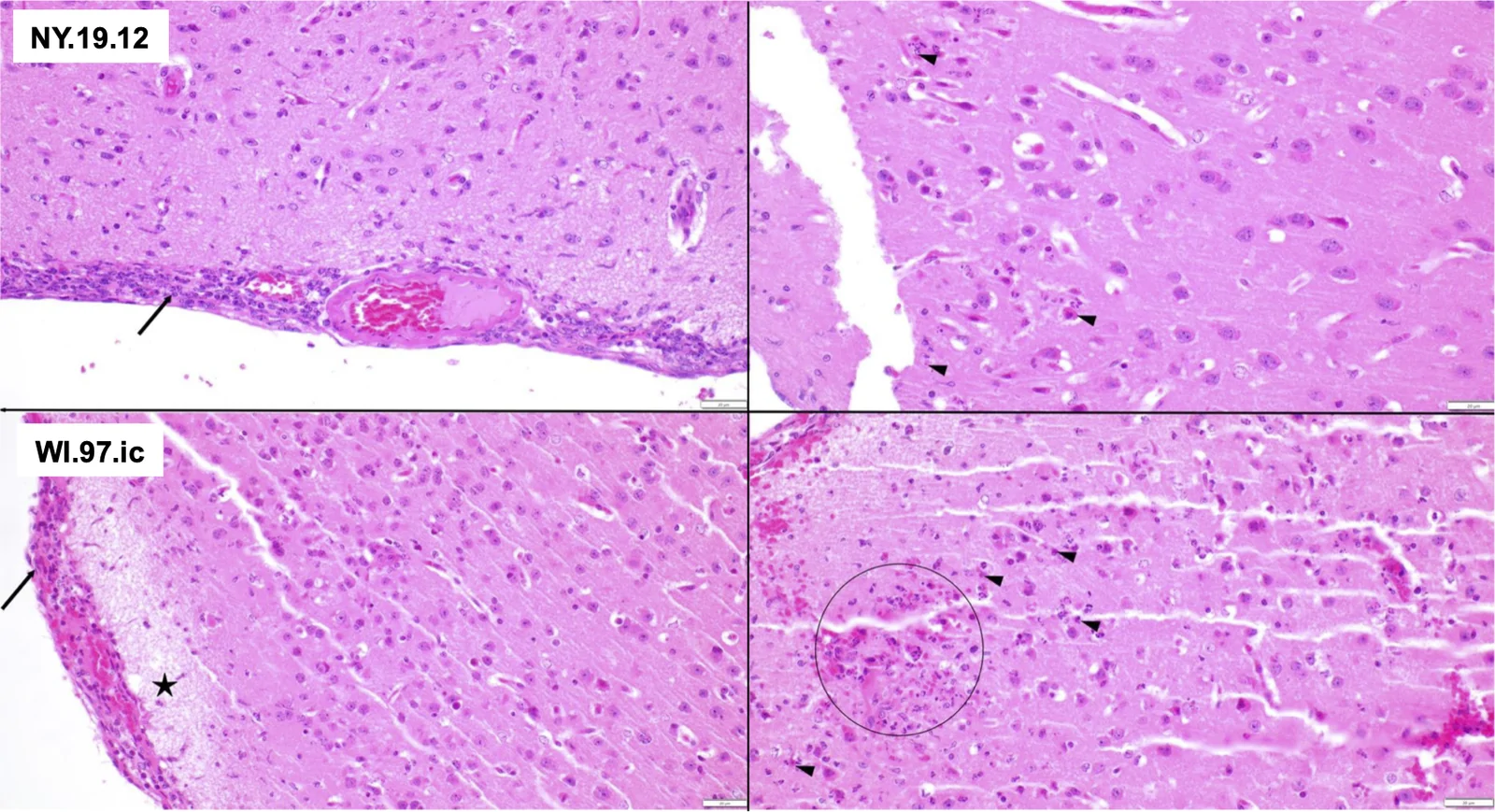
POWV-infected mouse brains showed similar severity and distribution of inflammation at disease endpoint. Representative histopathology of NY.19.12-infected (top) and WI.97.ic-infected (bottom) mouse brains. There is meningitis (arrows), superficial cerebral edema (star), neuronal degeneration and necrosis (arrow heads), and neutrophilic inflammation with hemorrhage (circle). Scale bars = 20 µm. H&E.

**Fig 4:**
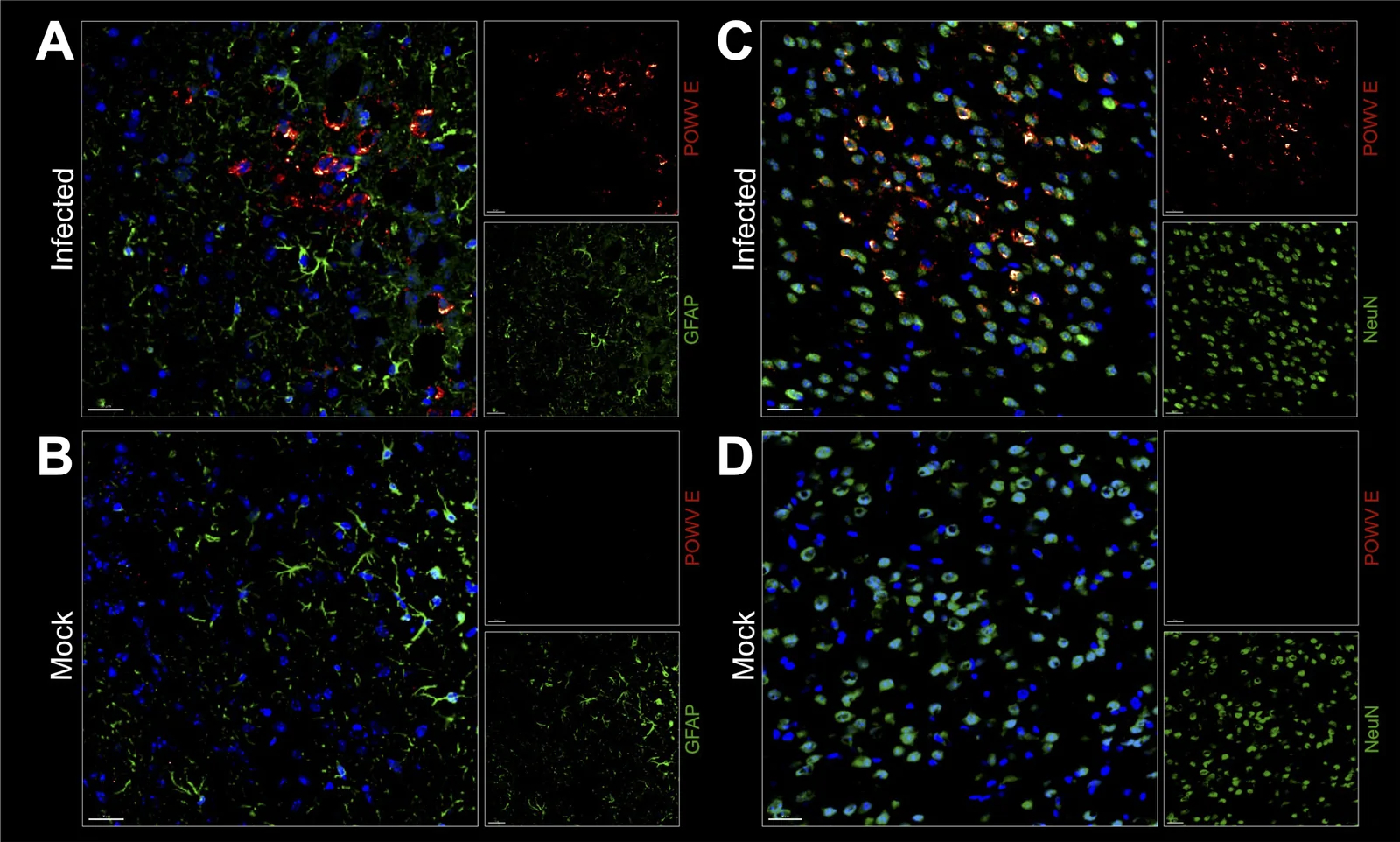
NY.19.12 specifically infects neurons with minimal astrocyte colocalization. Mice were S.C. inoculated with 10^3^ PFU NY.19.12 and sacrificed at 6 days post-infection, when infected brains were processed for IFA. Representative axial images show (**A**) an NY.19.12-infected mouse brain stained for astrocytes (GFAP) in green and POWV E in red, (**B**) an uninfected mock brain stained for GFAP and POWV E, (**C**) an NY.19.12-infected mouse brain stained for neurons (NeuN) in green and POWV E in red, and (**D**) an uninfected mock brain stained for NeuN and POWV E.

The right side of the brain from each mouse that succumbed to disease (i.e., met euthanasia criteria) at day 10 was characterized via histopathology. Overall, lesions in brains from both WI.97.ic and NY.19.12 were similar by disease endpoint (Fig. 3). In the cerebrum of all infected brains, there was lymphohistiocytic meningitis to meningoencephalitis with gliosis and neuronal degeneration and necrosis. To further characterize neurotropism of NY.19.12 at early time points, mice were S.C. inoculated with NY.19.12 and sacrificed at day 6 post-infection, where brains were processed for an immunofluorescence assay (IFA) targeting POWV, astrocytes (GFAP), and neurons (NeuN). Representative images show that there was minimal colocalization of NY.19.12 with astrocytes (Fig. 4A and B). However, colocalization of NY.19.12 with neuronal cells was observed (Fig. 4C and D).

### Genetic and phenotypic differences between strains

To determine whether the amino acid substitutions present in NY.19.12 were maintained post-neuroinvasion, POWV was sequenced from mice that were qRT-PCR positive for POWV vRNA in the cortex (Fig. 2H). All three substitutions (E-S205N, NS1-K178R, and NS5-R649K) were maintained post-neuroinvasion, and WI.97.ic did not acquire them (Table 2).

**TABLE 2:** Sequence of POWV in cortical tissue

Strain	Sample days	E		NS1		NS5	
		S205	N205	K178	R178	R649	K649
WI.97.ic	8, 8, 10, 10	100% (4/4)	0% (0/4)	100% (4/4)	0% (0/4)	100% (4/4)	0% (0/4)
NY.19.12	6, 7, 7, 10	0% (0/4)	100% (4/4)	0% (0/4)	100% (4/4)	0% (0/4)	100% (4/4)

**Fig 5:**
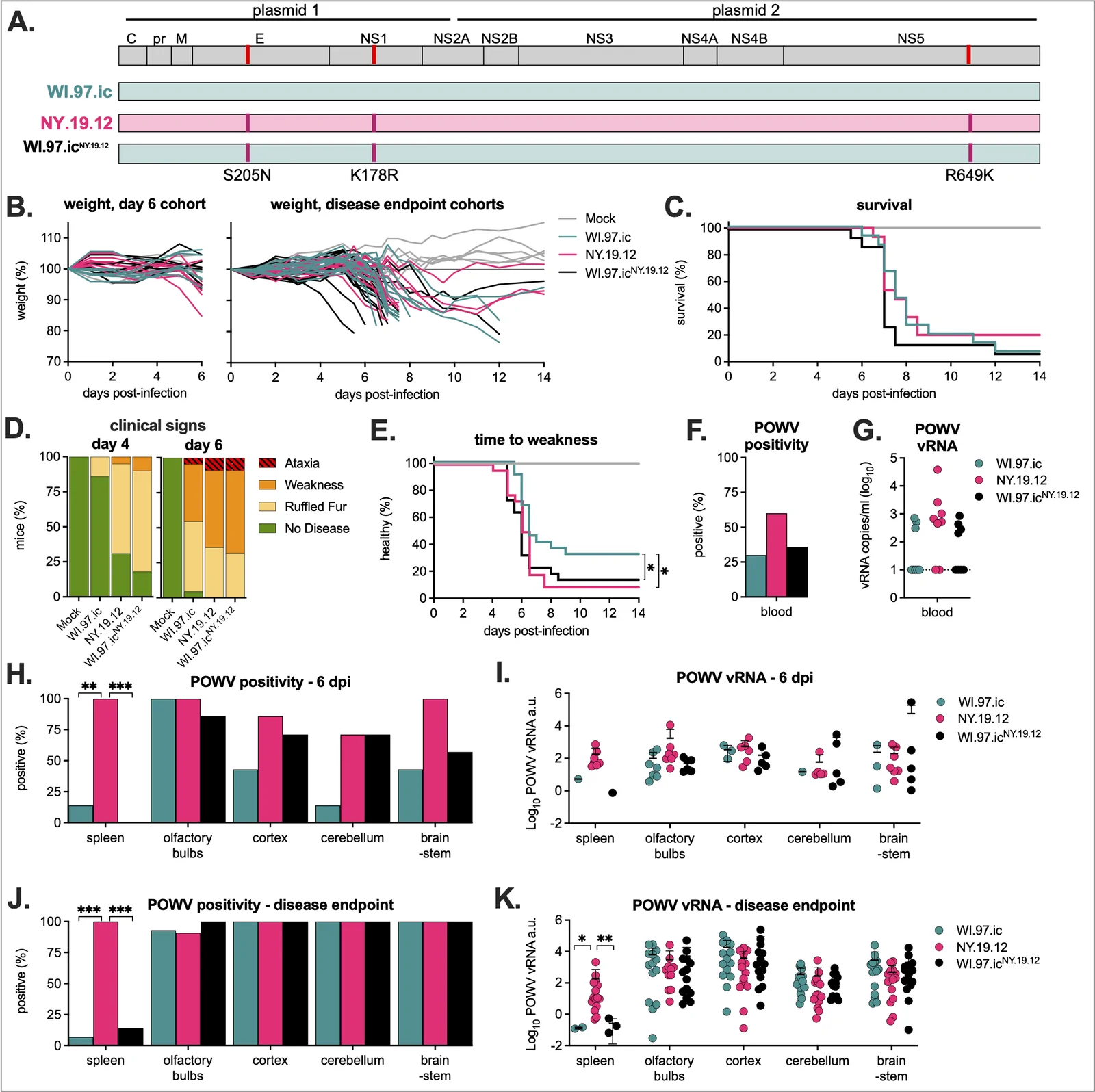
WI.97.ic^NY.19.12^ disease progression and virus distribution in the brain at 6 days post-infection and at disease endpoint. (**A**) NY.19.12 substitutions (E-S205N, NS1-K178R, and NS5-R649K) were engineered into a WI.97.ic two-plasmid backbone, producing a mutant infectious clone (WI.97.ic^NY.19.12^). Mice were S.C. inoculated with 10^3^ PFU POWV (WI.97.ic^NY.19.12^, NY.19.12, and WI.97.ic) or mock and sacrificed at either day 6 post-infection (*n* = 7 per strain) or at endpoint (*n* = 15 total, two independent experiments, *n* = 8 and *n* = 7 per strain). (**B**) Percent weight change from starting weight for day 6 cohort and endpoint cohorts. (**C**) Kaplan-Meier survival curves for disease endpoint cohorts with no significant differences between strains with log-rank (Mantel-Cox) test. (**D**) Clinical signs 4 and 6 days post-infection for all cohorts combined (*n* = 22 per strain). (**E**) The number of days post-infection for mice to exhibit weakness. Strains were compared using log-rank (Mantel-Cox) test, *P < 0.05. For blood samples from day 2 post-infection, (**F**) positive detection of POWV vRNA (%), and (**G**) vRNA levels (day 6 cohort, one endpoint cohort, *n* = 14 per strain). The dotted line shows the limit of detection. Positive detection of POWV vRNA (%) of (**H**) day 6 and (**J**) disease endpoint cohort in the spleen, olfactory bulbs, cortex, cerebellum, and brainstem. Fisher’s exact test, **P < 0.01, ***P < 0.001. Log_10_ POWV vRNA normalized to GAPDH intracellular levels from spleen, olfactory bulbs, cortex, cerebellum, and brainstem for (**I**) day 6 cohort and (**K**) disease endpoint cohorts (mean ± SD). Within each sample type, two-way ANOVA with Tukey’s multiple comparisons test, *P < 0.05, **P < 0.01. a.u. = arbitrary units. (mock = gray, teal = WI.97.ic, pink = NY.19.12, black = WI.97.ic^NY.19.12^).

To investigate the role of these amino acid substitutions during pathogenesis in mice, they were engineered into a WI.97.ic infectious clone backbone, designated WI.97.ic^NY.19.12^ (Fig. 5A). Morbidity, mortality, and tissue tropism were assessed for WI.97.ic^NY.19.12^ compared to NY.19.12 and WI.97.ic within the brain and spleen for two cohorts: 6 days post-infection and disease endpoint (i.e., individual mice meeting euthanasia criteria). Consistent with previous results (Fig. 2), there was minimal weight loss by day 6 post-infection, but by day 12 post-infection, mice in all cohorts had either lost weight and met criteria for euthanasia or gained weight and recovered (Fig. 5B). There were no significant differences in survival between strains (Fig. 5C), but at day 4 post-infection, ~70% of WI.97.ic^NY.19.12^- and NY.19.12-infected mice displayed mild clinical signs compared to less than 20% of WI.97.ic-infected mice (Fig. 5D). By day 6 post-infection, there were higher rates of neurologic clinical signs (weakness) in NY.19.12- and WI.97.ic^NY.19.12^-infected mice compared to WI.97.ic-infected mice (Fig. 5D). Additionally, the time it took mice to exhibit clinical signs of weakness was different, where there was significantly more weakness observed in NY.19.12 and WI.97.ic^NY.19.12^ cohorts compared to WI.97.ic-infected mice overall (*P* < 0.05) (Fig. 5E).

Although vRNA detection and levels in the blood were not statistically significant at 2 days post-infection, NY.19.12-infected mice showed trends toward higher rates of viremia and higher vRNA levels compared to both WI.97.ic and WI.97.ic^NY.19.12^ (Fig. 5F and G). By day 6 post-infection, vRNA was detected at comparable rates in the olfactory bulbs across strains (Fig. 5H). WI.97.ic^NY.19.12^ displayed an intermediate phenotype between NY.19.12 and WI.97.ic, with higher rates of vRNA detection in the cortex, cerebellum, and brainstem compared to WI.97.ic (Fig. 5H). Levels of vRNA within each brain tissue were comparable across strains (Fig. 5I). For the disease endpoint cohort, vRNA was detected at similar frequencies and levels in the olfactory bulbs, cortex, cerebellum, and brainstem samples across infection cohorts (Fig. 5J and K). Notably, compared to both WI.97.ic and WI.97.ic^NY.19.12^, vRNA was detected at significantly higher rates in the spleen of NY.19.12-infected mice at both day 6 post-infection and disease endpoint and had significantly higher levels of vRNA by disease endpoint (Fig. 5H, J and K).

**Fig 6:**
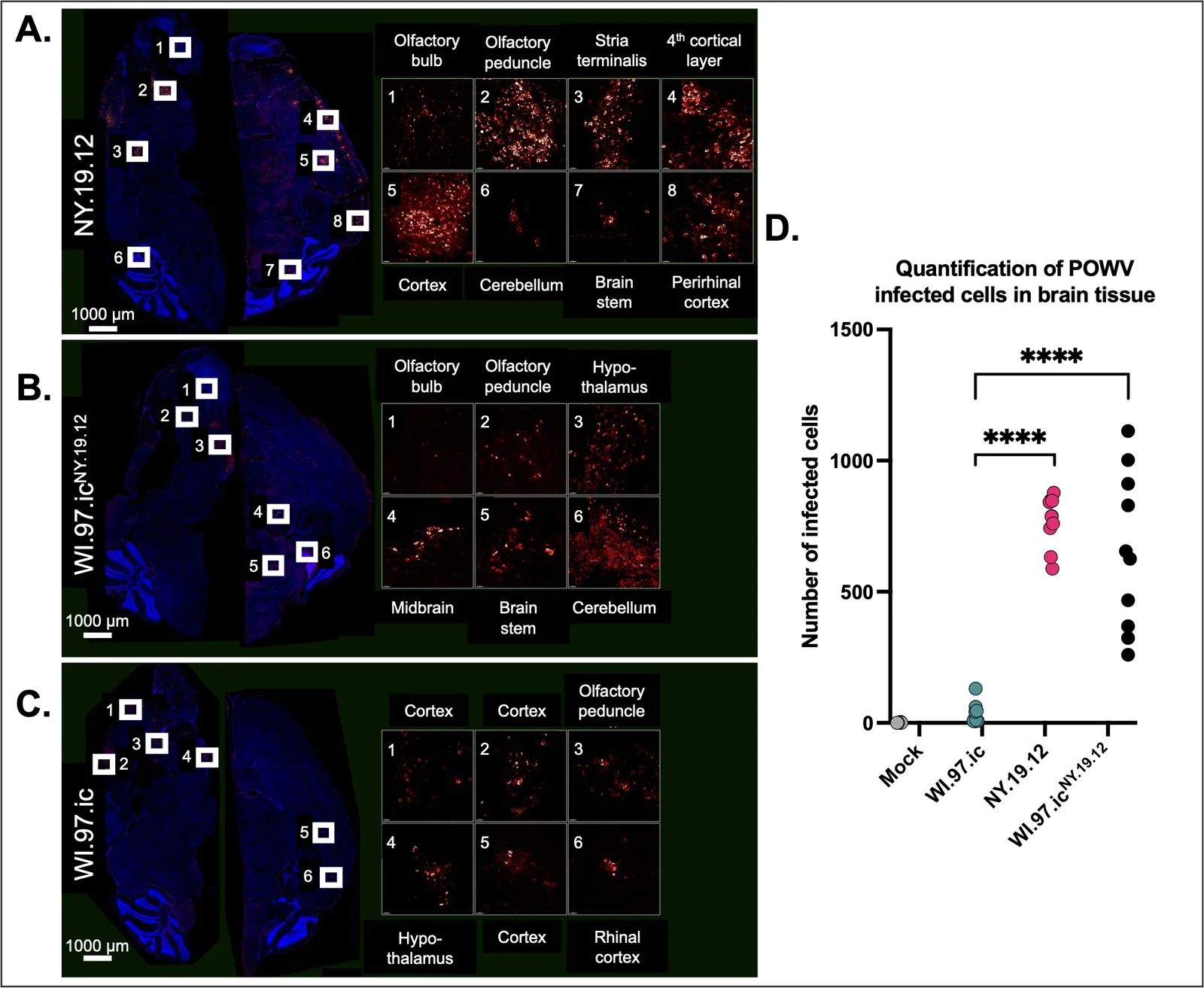
POWV viral distribution of NY.19.12-, WI.97.ic^NY.19.12^-, and WI.97.ic-infected mice in the brain. Mice were S.C. inoculated with 10^3^ PFU POWV (WI.97.ic^NY.19.12^, NY.19.12, and WI.97.ic) or mock and sacrificed at day 6 post-infection (*n* = 7 per strain). Brains were processed for IFA and stained for POWV antigen in red. Two representative sagittal images are shown per strain. White, zoomed-in boxes show detection of POWV antigen within specific brain regions. (**A**) NY.19.12-infected mouse brains with POWV detection in the (1) olfactory bulb, (2) olfactory peduncle, (3) stria terminalis, (4) 4th cortical layer, (5) cortex, (6) cerebellum, (7) brain stem, and (8) perirhinal cortex. (**B**) WI.97.ic^NY.19.12^-infected mouse brains with POWV detection in the (1) olfactory bulb, (2) olfactory peduncle, (3) hypothalamus, (4) midbrain, (5) brain stem, and (6) cerebellum. (**C**) WI.97.ic-infected mouse brains with POWV detection in the (1 and 2) cortex, (3) olfactory peduncle, (4) hypothalamus, (5) cortex, and (6) rhinal cortex. (**D**) Quantification of the number of infected cells in whole sagittal sections from mock, WI.97.ic-, NY.19.12-, and WI.97.ic^NY.19.12^-infected brain tissue, two brains per condition and 4–6 sections per brain (one-way ANOVA with Tukey’s multiple comparisons, ****P < 0.0001) (mock = gray, teal = WI.97.ic, pink = NY.19.12, black = WI.97.ic^NY.19.12^).

Infected mice brains were then processed for IFA and stained for POWV antigen (Fig. 6A through C). Representative images show that POWV antigen was detected in the olfactory bulbs, cerebellum, and brainstem of NY.19.12- and WI.97.ic^NY.19.12^-infected brains. However, WI.97.ic^NY.19.12^ and WI.97.ic had POWV infection in the hypothalamus. All infection groups shared POWV distribution patterns in the olfactory peduncle. The number of POWV-positive cells was quantified for mock, WI.97.ic^NY.19.12^-, NY.19.12-, and WI.97.ic-infected brain tissue, where NY.19.12 and WI.97.ic^NY.19.12^ had significantly higher amounts of POWV-infected cells compared to WI.97.ic (*P* < 0.0001) (Fig. 6D).

## DISCUSSION

We investigated the pathogenesis of a contemporary, low-passage POWV lineage II strain, NY.19.12, to assess the extent of strain-dependent variation in disease phenotypes relative to the historical Spooner-derived lineage II strain, represented by a rescued infectious clone (WI.97.ic) (17). POWV lineage II strains exhibit considerable genetic diversity and are an emerging cause of human neuroinvasive disease in North America; however, most experimental studies rely on the historical, highly passaged lineage I LB strain or lineage II Spooner strain, limiting our understanding of how naturally circulating viruses compare *in vivo* (13, 18–20). NY.19.12 was isolated in 2019 from *I. scapularis* in Saratoga County, New York, a region with documented human POWV cases in recent years (3, 9). NY.19.12 was sequenced and confirmed to be representative of currently circulating POWV strains in the Northeast United States.

Minimally passaged field isolates more accurately reflect circulating virus strains that may cause human disease compared to highly passaged laboratory strains. Prior work has shown variation in pathogenesis among lineage I strains and between lineage I and II POWV in mice, including differences in neurotropism and inflammatory profiles (16). However, the extent of phenotypic diversity within lineage II remains poorly defined, despite evidence of extensive genomic variability and reports that different lineage II isolates display variable replication and dissemination kinetics *in vitro* and *in vivo* (13, 18, 21–23). Our study revealed variation in morbidity and mortality in mice with some common features of pathogenesis that are consistent between strains. When 8-week-old mice were S.C. inoculated at 10^3^ PFU—a mouse age, route, and dose consistent with established POWV mouse models (11, 13, 14, 24)—both NY.19.12 and WI.97.ic caused weight loss and subsequent neurological disease. Regardless of strain, mice that met euthanasia criteria had high levels of vRNA detected throughout the brain, and histopathological evaluation of the brain showed a robust inflammatory response. These data suggest that core features of lineage II neuroinvasion are conserved across lineage II strains. Although C57BL/6 mice are widely used for POWV pathogenesis studies and allow direct comparison with prior work, host genetic background influences flavivirus susceptibility and neuroinvasion. The literature shows that it is possible that other mouse models such as C3H/HeJ, Collaborative Cross, or a more neurologically susceptible model could further resolve strain-dependent differences in pathogenesis or CNS tropism (19, 25, 26). Future studies evaluating additional mouse backgrounds or immune-modified models may help clarify these effects. Although no significant sex differences have been reported among POWV mouse models, both males and females should be used in future studies to address sex-specific differences (10, 16). Nonetheless, the C57BL/6 mouse model reproducibly captures key features of lineage II neuroinvasive disease and provides a consistent platform for comparative analysis.

Despite similarities in disease endpoint neuropathology between NY.19.12 and WI.97.ic, significant differences in disease kinetics were evident, where NY.19.12 induced a more rapid onset of clinical signs. Although vRNA levels in the serum and brain were not significantly different between groups, we observed trends toward earlier vRNA detection in mice infected with NY.19.12 compared to WI.97.ic. This suggests that the phenotypic differences observed during infection may reflect variation in the timing or frequency of viral dissemination rather than large differences in vRNA levels. These strain-dependent differences in clinical progression and tissue tropism demonstrate the variation within lineage II pathogenesis. Consistent with current POWV and tick-borne flavivirus literature, NY.19.12 infection primarily targeted neurons, with limited astrocyte colocalization—a pattern associated with severe neurological sequelae (15, 16, 27). Although some studies have characterized neuropathology and immune involvement in the CNS following POWV infection, detailed understanding of cell type-specific responses and regional viral spread is limited (21, 27). Recent work with whole-brain imaging in related tick-borne flaviviruses has demonstrated that structural proteins and nonstructural components can influence patterns of neuroinvasion, supporting the need for high-resolution mapping of viral and host to investigate POWV neurovirulence and CNS tropism (28, 29).

Our analysis identified three amino acid substitutions shared by NY.19.12 and phylogenetically related lineage II viruses in the E, NS1, and NS5 proteins. The E protein residue (S205N) occurs in domain II, a region involved in membrane fusion and structural rearrangements required for viral entry. The NS1 residue (K178R) lies within the β-ladder domain, which contributes to NS1 dimerization and interactions with membranes and other viral proteins during replication complex formation. The NS5 residue (R649K) is located within the RNA-dependent RNA polymerase domain, which is responsible for viral genome replication. All the mutations represent conservative substitutions between positively charged residues; however, the E-S205N substitution introduces a larger side chain within a structurally important region of the E protein. Notably, these amino acid substitutions were not detected in the brains of WI.97.ic-infected mice that succumbed to disease (i.e., met euthanasia criteria), suggesting that they are unique to NY.19.12 and may contribute to viral distribution in the brain. Together, these amino acids represent potential molecular differences and likely confer a fitness advantage, as evidenced by their maintenance in nature.

By using WI.97.ic as a lineage II reference strain, we aimed to not only define strain-specific features of lineage II pathogenesis but also identify viral determinants underlying these differences by directly genetically manipulating the infectious clone. The E, NS1, and NS5 amino acids present in NY.19.12 were engineered into the clone and rescued as infectious virus (WI.97.ic^NY.19.12^). When mice were infected with WI.97.ic^NY.19.12^, comparable cerebellar vRNA levels, similar distribution of infection in multiple brain regions, and a similar quantity of infected cells in brain tissue relative to the natural NY.19.12 isolate were observed, suggesting that these residues contribute to distinct neurotropism at early time points. While vRNA detection provides a sensitive measure of POWV RNA in sectioned brain homogenates, IFA-based infected cell quantification adds spatial and cellular resolution by identifying antigen-positive cells within defined brain regions. Together, these complementary approaches provide a more detailed assessment of CNS infection, capturing both vRNA detection and the regional distribution of infected cells. However, the mechanisms by which these residues influence neurotropism, whether through altered receptor engagement, immune evasion, or replication kinetics, remain to be elucidated (30, 31).

Importantly, certain phenotypes observed with NY.19.12 were not recapitulated by WI.97.ic^NY.19.12^, particularly high vRNA levels and persistence in the spleen, suggesting that determinants outside the engineered mutations contribute to peripheral tropism. The spleen has been implicated as a site of replication for flaviviruses including dengue virus and POWV, where the infection of macrophage populations may influence systemic spread and immune activation (15, 32, 33). In addition, high viral load and persistence in the spleen have been correlated to POWV lethality in mice (34). Our group has shown that multiple lineage II isolates exhibit robust spleen infection early post-inoculation, indicating that spleen tropism may not be unique to NY.19.12 (18). Whether this reflects quasispecies diversity, non-coding genomic elements, or other polymorphisms remains unclear, as infectious clones such as WI.97.ic may lack the population diversity found in natural isolates and often exhibit attenuated phenotypes in peripheral tissues (31, 35).

Beyond nonsynonymous coding changes, other genetic factors may influence strain-specific pathogenesis. Synonymous mutations can alter RNA secondary structure, untranslated region variants can modulate replication efficiency or translation, and individual single nucleotide polymorphisms have been linked to virulence differences in other flaviviruses such as Japanese encephalitis virus and tick-borne encephalitis virus (36–40). Moreover, non-coding RNAs and host-pathogen regulatory interactions may also shape susceptibility and disease outcomes (39). Incorporating transcriptomic and epigenomic analyses in future studies will be essential to understand how these factors contribute to POWV pathogenic variation.

Our findings demonstrate that key features of lineage II disease are maintained in C57BL/6 mice, but that disease course and tissue tropism vary substantially between strains. Amino acid differences in both structural and nonstructural proteins likely contribute to these phenotypic differences, although the influence of other viral determinants and the extent to which natural strain variation affects disease severity in humans remain unclear. Together, these results highlight the importance of examining minimally passaged field isolates to capture the biological diversity of POWV lineage II and better understand its implications for transmission and pathogenesis.

## MATERIALS AND METHODS

### Cells and viruses

BHK-21 (ATCC CCL-10) and HEK293T (ATCC CRL-3216) cells were grown in Dulbecco’s modified Eagle medium (DMEM) supplemented with 10% fetal bovine serum (FBS), 10 units/mL penicillin, and 10 μg/mL streptomycin at 37°C and 5% CO_2_. Viruses (described in Table 1) were passaged on BHK cells; the supernatant was harvested 3–6 days post-infection and frozen at −80°C prior to use. Virus inoculum for mouse studies was backtitered via plaque assays to validate virus concentration. All virus work was conducted in Colorado State University BSL-3 containment.

### Plaque assays

Standard plaque assays were used to determine infectious virus concentrations. BHK cells were seeded 1 day prior to infection to be confluent the following day. Virus samples were thawed, serially diluted in infection media (standard growth media with 2% FBS) in duplicate, added to cells, and incubated at room temperature for 1 h on a plate rocker. A POWV strain with a known titer was used as a positive control. Cells were overlaid with semi-solid 0.6% tragacanth medium and incubated at 37°C for 5 days. Cells were fixed and stained with 20% ethanol (EtOH) and 0.1% crystal violet. Plaques were counted manually to calculate titer.

### POWV mouse infections

C57BL/6 mice (strain #000664) were obtained from Jackson Laboratories. Approval for animal protocols was obtained from the Colorado State University Institutional Animal Care and Use Committee (protocol #5074). All animal infections were conducted in Colorado State University ABSL-3 containment. Eight-week-old female mice were S.C. inoculated at the inguinal region with 10^3^ PFU POWV or mock-infected with 100 µL DMEM. Prior POWV mouse studies have commonly used footpad S.C. inoculation to model peripheral infection; however, in this study, the inguinal region was selected as a defined S.C. site to facilitate evaluation of viral dissemination from the inoculation site to the draining inguinal lymph node, peripheral tissues, and CNS. This method was chosen to maintain consistency across POWV studies in our group and has been used in the literature with other animal models (23). Mice were weighed, and clinical signs were recorded unblinded by the same individual throughout the course of the studies. Food pellets were placed at the bottom of the cage once clinical sign onset was observed. Clinical sign criteria were categorized as follows: healthy, hunched/ruffled fur (mild), weakness (moderate), and ataxia. Forelimb weakness was assessed by a grip test, and hindlimb weakness was observable by eye. Disease endpoint indicates that mice met euthanasia criteria, where clinical signs of ataxia were observed or when mice lost 20% of their starting weight.

### Mouse necropsies and sample processing

Submandibular blood collection was performed using 4-mm lancets, and blood was stored in BD Microtainer tubes with serum separator additive (serum) or EDTA (whole blood). For necropsies, mice were euthanized via 5% isoflurane and cervical dislocation, and blood was collected via cardiac puncture. Cortex and spleens were harvested. For brain region analysis using immunofluorescence, mice were deeply anesthetized with 5% isoflurane and perfused with 30 mL of PBS, then 30 mL of freshly prepared 4% paraformaldehyde, and brain regions were harvested. For brain region analysis via qRT-PCR, mice were deeply anesthetized with 5% isoflurane and perfused with 30 mL of PBS, and brain regions were harvested. For the infection kinetics study (Fig. 2), samples were placed into 2 mL snap cap tubes with a metal bead, and 10% of each tissue weight was added in volume with DMEM and frozen at −80°C until homogenization. For subsequent studies, samples were placed into 2 mL snap cap tubes with 1 mL of 1× DMEM and a metal bead and frozen at −80°C until homogenization. Mouse tissues were rapidly thawed and homogenized at 30 Hz/s for 2 min and then centrifuged at maximum speed for 10 min to pellet cellular debris.

### Nucleic acid extraction and qRT-PCR

Viral nucleic acid was extracted from 50 μL of serum, whole blood, or homogenized mouse tissue using the Omega Viral DNA/RNA Extraction Kit on the KingFisher Flex instrument following manufacturer’s instructions. Samples were either normalized for qRT-PCR via 10% wt/vol per sample before homogenization (Fig. 2) or to total RNA concentration after homogenization (Fig. 5). Quantitative real-time PCR (qRT-PCR) was performed using the EXPRESS One-Step qRT-PCR Kit and POWV primer-probes targeting the NS5 coding region (blood, spleen, olfactory bulbs, cortex, cerebellum, and brainstem) and EXPRESS One-Step SYBR GreenER Kit with mouse GAPDH primers (spleen, olfactory bulbs, cortex, cerebellum, and brainstem) in duplicate (Table 3) (41). POWV RNA standards, covering the NS5 gene as previously described, were used as a positive control for all POWV qRT-PCR. For Fig. 2 and blood samples, vRNA copies were determined using the RNA standard and reported as genome copies per mL or per gram of tissue. For Fig. 5, the values were reported as fold change normalized to GAPDH using the ΔΔCT method (18). These data sets were analyzed within each experiment and were not directly compared across reporting methods. Details are provided in figure legends.

**TABLE 3:** Primer-probe sequences for qRT-PCR assays

Primer	Sequence (5’ → 3’)
POWV forward	GGCCATGACAGACACAACA
POWV reverse	AGCCAGGCACCAGGGTGATCAT
POWV probe	CACATCCTCACCCTGGCTC (FAM)
Mouse GAPDH forward	TGTGTCCGTCGTGGATCTGA
Mouse GAPDH reverse	CCTGCTTCACCACCTTCTTGA

### Sanger sequencing of mutations

vRNA was extracted as described above, and reverse transcription and PCR were performed using QIAGEN OneStep RT-PCR Kit following the manufacturer’s protocol and primers to amplify regions containing each of the mutations (E, NS1, and NS5) (Table 4). PCR products were gel purified and cleaned up using MACHERRY-NAGEL NucleoSpin Gel and PCR Clean-up kit, then Sanger sequenced.

**TABLE 4:** Primer sequences for Sanger sequencing

Primer	Sequence (5′ → 3′)
E forward	CTGCAAATGAGACCAATGAGAAC
E reverse	CTCCACAAGTTTCTCCACACT
NS1 forward	TTGTGGAGTGTGCCTGAAA
NS1 reverse	AGTGAGAATGAGCTCCTGAATG
NS5 forward	TGGAAGGTGAGGGAGTCAT
NS5 reverse	CCACATCCTTACGGGTCTTT

### Generation of mutant infectious clones

The POWV lineage II two-plasmid cDNA infectious clone (WI.97.ic) was obtained from Aaron Brault, U.S. Centers for Disease Control and Prevention (CDC), and was rescued and propagated as previously described (17). E and NS1 mutations were engineered into plasmid 1 via NEBuilder HiFi DNA Assembly Cloning Kit, and the NS5 mutation was engineered into plasmid 2 via NEB Q5 Site-Directed Mutagenesis Kit, using primers designed via NEBuilder Assembly Tool. Mutant infectious clone (WI.97.ic^NY.19.12^) plasmids were transformed into Stbl3 *Escherichia coli* and grown on ampicillin-treated agar plates at 37°C overnight. Individual colonies were isolated, grown overnight in liquid culture, and purified via QIAprep Spin Miniprep Kit. Whole plasmids were sequence verified using Plasmidsaurus. Plasmids 1 and 2 were linearized with BstZ171 and ligated together at a 1:3 ratio with T4 DNA ligase. The ligated product was linearized with NotI, and RNA transcribed via mMESSAGE mMACHINE T7 Transcription Kit. Full-length WI.97.ic^NY.19.12^ RNA was transfected into HEK293T cells using Lipofectamine 3000. The supernatant was collected at approximately 4–5 days post-transfection, once the cells reached ~50% cytopathic effect. Viral stocks were passaged on BHK-21 cells, concentrated via centrifugation, and titered by plaque assay. Virus sequence at each mutation site (E, NS1, and NS5) was confirmed via Sanger sequencing as described above.

### Histopathology

Mouse heads were fixed in 10% paraformaldehyde and then decalcified for 48 h in nitric acid. Sagittal sections of the skull were then paraffin-embedded, routinely processed, and stained with hematoxylin and eosin (H&E) for histologic evaluation. Pathologists were blinded to treatment groups.

### Immunohistochemistry

The right hemisphere from the brains of the infected mice was dehydrated in 30% sucrose, frozen in OCT Embedding matrix, and sectioned (sagittal and axial sections) at 10 μm thickness using a CryoStar NX50 cryostat according to the manufacturer’s instructions at −20°C. Slides were thawed at 37°C for 5 min and hydrated in PBS, followed by permeabilization and blocking using PBS, 0.2% Triton X, 10% goat serum, and 0.1% NaN3. Sections were stained with primary antibodies against POWV E protein (GeneTex, GTX640340, 1:1,000), POWV NS3 protein (GeneTex, GTX642219, 1:1,000), astrocyte marker GFAP (Abcam, ab4674, 1:500), and secondary antibodies donkey anti-rabbit A488 (Invitrogen, A21206, 1:500), donkey anti-rabbit A647 (Invitrogen, A31573, 1:500), DAPI (Sigma, 1:1,000), and neuronal marker NeuN-A647 (Abcam ab190565, 1:200). Slides were mounted and imaged by Leica SP8 confocal microscope using a HC PL APO 20x/0.75 Dry objective. Images were created using Imaris software (version 10.1.1, Bitplane, Zürich, Switzerland). For POWV antigen quantification, axial brain slices from half a brain (two mice/virus and two mock-infected mice, 4–6 sections per animal) were stained with POWV NS3 (GeneTex, GTX642219) and scanned using the Pannoramic 250 Flash III digital slide scanner (3DHISTECH Ltd., Budapest, Hungary), and the number of positive cells was quantified using the “Spots” tool in Imaris (v. 10.1.1) with a cell diameter set at 15 μm. The total number of infected cells/brain section was plotted using GraphPad Prism v10.6.1 (GraphPad Software Inc., San Diego, CA, USA).

### Data analysis and statistics

All data were analyzed using GraphPad Prism version 9.5.0. Statistical tests are described in figure legends. Sequences were analyzed in Geneious Prime 2026.0.2.

## Data Availability

All data are available upon request.
